# Mapping the type, frequency, intensity, temporality, and pathways of dissemination strategies during the national scale-up of *TransformUs Secondary*

**DOI:** 10.1093/tbm/ibaf089

**Published:** 2026-01-13

**Authors:** Anna Fitriani, Harriet Koorts, Ana María Contardo Ayala, Natalie Lander, Jess Orr, Nicole Martin-Alcaide, Jo Salmon

**Affiliations:** Institute for Physical Activity and Nutrition, School of Exercise and Nutrition Sciences, Deakin University, Geelong, VIC, Australia; Institute for Physical Activity and Nutrition, School of Exercise and Nutrition Sciences, Deakin University, Geelong, VIC, Australia; Institute for Physical Activity and Nutrition, School of Exercise and Nutrition Sciences, Deakin University, Geelong, VIC, Australia; Institute for Physical Activity and Nutrition, School of Exercise and Nutrition Sciences, Deakin University, Geelong, VIC, Australia; Institute for Physical Activity and Nutrition, School of Exercise and Nutrition Sciences, Deakin University, Geelong, VIC, Australia; Institute for Physical Activity and Nutrition, School of Exercise and Nutrition Sciences, Deakin University, Geelong, VIC, Australia

**Keywords:** dissemination strategies, physical activity, sedentary behavior, national scale-up, school-based intervention

## Abstract

**Background:**

Schools are ideal settings for implementing evidence-based physical activity interventions at scale due to their wide reach. However, dissemination strategies used to achieve scale are rarely reported.

**Purpose:**

This study aimed to describe the strategy type, frequency, intensity, temporality, and pathways used in disseminating the *TransformUs Secondary* initiative across Australia over the first 16 months of national scale-up.

**Methods:**

*TransformUs Secondary* is a whole-of-school initiative that targets behavioral, environmental, and pedagogical strategies inside and outside the classroom to reduce sedentary behavior and increase physical activity among adolescents aged 12–18 years. Since October 2023, the *TransformUs* team and 16 partner organizations collaboratively disseminated the initiative nationally. A dissemination activity log was used to record dissemination strategies, which were subsequently mapped *post hoc* to existing frameworks to categorize type, frequency, intensity, temporality, and pathways. Data are reported descriptively and graphically.

**Results:**

Between October 2023 and February 2025, 10 discrete strategies were identified, with the most frequent and intensive strategies being “Promotion via mass media” (33.2%, 110 person-hours), “Develop educational materials” (20.8%, 48 person-hours), and “Maintain partner engagement” (12.4%, 20 person-hours). Strategy frequency and intensity fluctuated and increased after the initiative launch, shifting the focus from targeting partner organizations to targeting school staff. Notably, 54.3% of dissemination strategies occurred via direct pathways to school staff.

**Conclusion:**

Reporting dissemination strategies and pathways clarifies how school-based interventions are scaled in practice, providing evidence to inform research, guide policy, and support effective implementation in schools.

**Clinical Trial information:**

The Clinical Trials Registration #ACTRN12622000600741.

## Graphical abstract

**Graphical Abstract:**
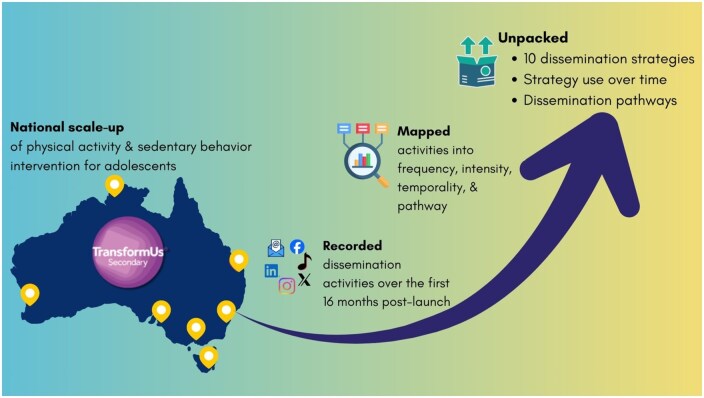

Implications **Practice:** Implementation of school-based physical activity initiatives should prioritize dissemination via tailored messages and channels from key education and health partners to maximize reach and adoption.

**Policy:** Policy makers, funders, and decision-makers should invest in sustained, user-focused and policy relevant dissemination approaches to accelerate national scale-up of school-based physical activity programs.

**Research:** Researchers should investigate how diverse dissemination strategies influence adoption and implementation outcomes and uncover mechanisms driving successful dissemination over time.

## Introduction

The World Health Organization proposes for whole-of-school approaches to tackle low levels of physical activity and high levels of sedentary behavior among children and adolescents [1]. Schools provide an ideal setting for such interventions, offering a structured environment to promote physical activity and reduce sitting time during classes, recess, and extracurricular programs [2]. However, the impacts of these interventions at population-level are often small or inconsistent [3–5], and few interventions become school routine nationally [2]. It has been suggested that raising awareness and motivating adoption is an essential first stage in scaling programs [6]. However, dissemination efforts are often overlooked in favor of focusing on program delivery [7]. These gaps highlight the need for interventions that are not only well-designed but also strategically disseminated to achieve meaningful uptake and population-level impact.

Scaling effective whole-school programs presents challenges due to heterogeneous school environments, staff autonomy, and multiple target audiences [8, 9]. The target audiences may include education departments, principals, teachers, support staff, health professionals, parents, and students [8]. Addressing these complexities requires tailored dissemination strategies to support program adoption [10]. Dissemination strategies (i.e. “any action or set of actions that target public health and healthcare decision-makers’, clinicians’, and other staffs’ awareness, knowledge, attitudes, and intention to adopt an evidence-based intervention”) are a critical element of achieving widespread reach and adoption of evidence-based interventions [11]. Examples include media development, mailings, mass media campaigns, presentations, and webinars [11]. Type, frequency, and channels of dissemination strategies often evolve during scale-up [12], highlighting the need to understand dissemination strategies and pathways to optimize dissemination in real-world school settings. Several approaches have been proposed to enhance this understanding, such as the development of the School Implementation Strategies, Translating Expert Recommendations for Implementing Change Resources (SISTER) taxonomy [13], the use of standardized frameworks to specify dissemination and implementation strategies (e.g. strategy name, actor, action, target, intensity, temporality, justification, and outcomes) [14], and clear documentation of dissemination pathways [6].

While whole-of-school programs targeting adolescent physical activity have been implemented in Australia (e.g. Physical Activity for Everyone/PA4E1), reporting has primarily focused on implementation outcomes such as student physical activity [15], fidelity, and reach within the context of randomized controlled trials [16], rather than real-world dissemination strategies. These studies typically examine program delivery, rather than the processes used to achieve state- or national-scale dissemination. Furthermore, a systematic review of strategies used in whole-of-school programs targeting health behavior, including physical activity and sedentary behavior, found that most of the strategies reported focused on implementation, not on disseminating programs at scale [7]. Thus, documenting dissemination strategies employed by the research team at a national level is critical for understanding how evidence-based programs can be effectively scaled in diverse real-world contexts.

*TransformUs* is an efficacious, whole-of-school initiative designed to enhance physical activity and reduce sedentary behavior among primary school children, integrating movement into core subjects (e.g. math, science, and English) as well as introducing environmental changes inside and outside the classroom [17]. The primary initiative has been part of a state-wide trial in Victoria, Australia, to assess implementation and effectiveness at scale; however, dissemination strategies used to achieve broader reach were not explicitly reported [18]. In 2022, *TransformUs* was adapted for the secondary school context (*TransformUs Secondary*), and is currently being tested at scale [19]. This study aimed to describe the type, frequency, intensity, and temporality of activities and pathways used by the research team in the dissemination of the *TransformUs Secondary* initiative across Australia over a 16-month period of national scale-up. Understanding these dissemination strategies is important for informing future efforts to scale school-based interventions effectively.

## Methods

### Design and setting

This study used data from the national implementation trial of the *TransformUs Secondary* initiative, currently being tested in a Type 2 hybrid implementation–effectiveness trial [19]. The initiative is specifically designed for secondary school students (aged 12–18 years) to integrate movement into the school day [19]. Its goals include promoting physical activity, reducing sedentary behavior, and supporting classroom engagement, focus, concentration, and self-regulation through embodied, experiential, or incidental learning [19]. It includes six core components: active academic lessons, active breaks, active homework, active school environments, health education lessons, and peer support [19]. The *TransformUs* website (https://transformus.com.au/) offers free professional learning and resources designed to help school staff implement the initiative.

Dissemination was strategically rolled out nationwide across all six states and two territories, targeting ∼1453 secondary schools, 239 169 staff, and 1 861 765 students [19]. This included government, Catholic, and independent schools, encompassing standalone secondary schools as well as combined (primary–secondary) and special school settings, which together represent the full secondary education system in Australia. During the first 16 months postlaunch (10 October 2023 to 10 February 2025), the program registered 185 secondary school staff from 124 schools across all states and territories, representing ∼9% of eligible secondary schools nationwide. Dissemination began in October 2023 with launches in South Australia and Queensland, consisting of online webinars with key partner organizations (e.g. state departments of education and health, principal and teacher professional associations) to introduce *TransformUs Secondary* and inform them that the initiative was now available for adolescents in secondary schools. Subsequent launches occurred in Western Australia and Northern Territory (November 2023), and Victoria, New South Wales, Australian Capital Territory, and Tasmania (March 2024). Since then, the *TransformUs* team and 16 partner organizations (from education, health, and active recreation sectors as described above) have been actively disseminating the initiative across all Australian states and territories.

Drawing on lessons learned from the dissemination of *TransformUs Primary* in Victoria [18] (e.g. engaging key stakeholders and using multiple communication channels), the team developed a dissemination plan for *TransformUs Secondary* that leveraged existing networks and strategies. This plan involved engaging partner organizations to extend initiative reach and creating a dissemination toolkit containing adaptable materials, including templates for social media posts and newsletters to support consistent and scalable promotion of the initiative. The team also used its own social media platforms to support broader awareness and engagement. The dissemination team comprised seven members with defined roles. While each had primary responsibilities, they often collaborated across activities based on expertise and capacity. The team covered four divisions: website management (*n* = 1), partner engagement (*n* = 2), teacher resource development (*n* = 1), and media development (*n* = 5). Two members of the media team were also members of other divisions, with one in partner engagement and another in website management.

### Data collection: tracking dissemination activities

An activity log (online Excel file) was used to track dissemination activities carried out by the *TransformUs* team (Supplementary File 1). The activity log was developed to enable tracking and collection of detailed descriptions of dissemination activities (e.g. message development, partner engagement meetings, presentations) [20]. The log collected the following information: activity name, purpose, duration, team member/s involved, target audience/attendees, details of activity, media/channel used, message delivered (y/n), output (if any). The log was distributed by email to *TransformUs* team members fortnightly, who were asked to record their dissemination activities in the previous 2 weeks over the 16-month dissemination period (3 October 2023–10 February 2025). Five team members contributed to completing the log. Each member was assigned to one of four divisions: website management, partner engagement, media, and teacher resource development, with partner engagement covered by two members. All members were responsive, and the logs were completed for all reporting periods. Where log entries were incomplete, the lead author contacted relevant team members to request clarification.

### Data management

#### Classifying activities into dissemination strategy type, frequency, and intensity

Dissemination activities collected from individual logs were consolidated and reviewed. Following previous work [20], “activities” were defined as the specific actions undertaken (e.g. sending emails, partner engagement meetings, developing materials), and “strategies” were defined as broader categories used to classify these activities based on their underlying function (e.g. distribute a promotional message, inform a local opinion leader). Because some activities served multiple functions, they were often mapped to more than one strategy based on the purpose and details recorded in the activity log. For example, a follow-up meeting with a partner organization representative after the launch, in which a dissemination toolkit was provided, was mapped to two strategies: “Maintain partner engagement” and “Distribute dissemination toolkit.” Supplementary File 2 presents a list of discrete strategies, with examples of unique dissemination activities mapped to each discrete strategy, and a notation indicating which framework (described below) was used to classify the strategies. Frequency was calculated based on the number of times each discrete strategy was assigned.

All recorded activities were coded using Leeman *et al.*’s classification framework [11] and the SISTER taxonomy [13], which adapts the Expert Recommendations for Implementing Change (ERIC) strategies for school-based contexts. Although the SISTER taxonomy is primarily used for mapping implementation strategies, some strategies, such as “Engage consumers,” and “Use mass media” were considered relevant for dissemination, and supplemented aspects that Leeman *et al.*’s classification might not fully capture. This approach has been applied in a prior study [21], where dissemination strategies for a national medical program were mapped using the ERIC [22] and the Leeman *et al.* classification framework [11].

The intensity of each discrete strategy was measured in person-hours. First, for each activity, the number of people involved was multiplied by the time spent (in hours) to calculate activity-level intensity. These person-hours were then summed across all activities assigned to each strategy to obtain the total intensity per discrete strategy. If one activity was mapped to several discrete strategies, the intensity of that activity was fully assigned to each corresponding strategy. If a 1-h webinar involved two team members, the activity-level intensity was calculated as 2 people × 1 h = 2 person-hours. If the activity was mapped to two discrete strategies, such as “Conduct teacher educational meetings” and “Distribute educational materials,” the full intensity (2 person-hours) was assigned to each strategy. Person-hours were mapped in full to each strategy and some activities overlapped across strategies, the sum of total person-hours across all strategies is therefore greater than the actual total in real time.

#### Temporality: strategy type, frequency, and intensity over time

Mapping strategy type, frequency, and intensity over time illustrates how dissemination efforts evolve in response to key events and the school calendar, providing insights for optimizing timing and targeting future scale-up activities. For temporality, the type, frequency, and intensity of discrete and overarching strategies were plotted fortnightly on a 16-month timeline, aligned with key events such as state launches and the Australian school calendar (consisting of four terms per year, each ∼10 weeks long, with 2-week breaks between terms, and a 6-week summer/end of year holiday). Because school term dates differed between states and territories, each school term includes the full range from the earliest start date to the latest end date across all states and territories.

#### Mapping dissemination strategies into dissemination pathways

Dissemination strategies were mapped according to Brownson *et al.*’s Dissemination Model [6], which includes source, channel, message, and audience (Supplementary File 2). To address audience segmentation, concepts from the Interactive Systems Framework [23] were included, distinguishing between the *TransformUs* team as the dissemination source (positioned as the synthesis and translation system), partner organizations as the support system audience, and school staff as the delivery system audience. Dissemination pathways were defined as either direct (*TransformUs* team to school staff—Delivery System) or indirect (*TransformUs* team to partner organizations—Support System- who then facilitated dissemination to school staff). Strategies that focused on outreach and audience engagement were classified as “engagement strategies” and grouped according to whether they targeted partner organizations within the support system or school staff within the delivery system. Strategies used solely for preparation before reaching any audience, such as development strategies, were excluded from this analysis.

### Analysis

All data were assigned to corresponding dates in a time series dataset. Qualitative labels of each activity were deductively mapped to Leeman *et al.*’s dissemination classification framework [11] and the SISTER taxonomy [13] to categorize strategies by their underlying function. Frequency and intensity of discrete strategies were analyzed using univariate techniques. Percentages of total frequency were calculated, and descriptive statistics (median, minimum, maximum) were computed for people involved and hours spent in dissemination strategies. Fortnightly frequencies and intensities of strategies were plotted. All statistical analyses were conducted using Stata SE 18.0 (StataCorp LLC, College Station, TX, United States).

## Results

### Dissemination strategies type, frequency, and intensity over the first 16 months

**Figure 1.**
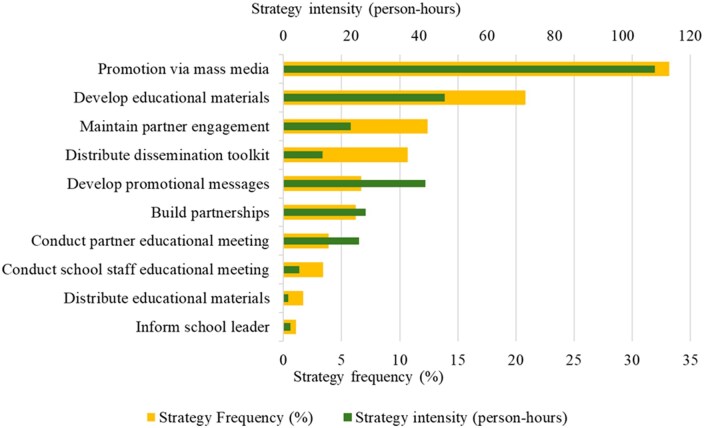
Frequency and intensity of dissemination strategies coded from 151 unique activities during the first 16 months postlaunch. Percentages indicate that the proportion of times each strategy was identified across all strategies coded.

Over time, the *TransformUs* team documented 151 unique dissemination activities, from which dissemination strategies were coded a total of 178 times. These activities were classified into 10 discrete strategies (Fig. 1). The most frequently used strategies aimed to raise school staff awareness and encourage engagement with the program. “Promotion via mass media” accounted for 33.2% of all strategies and aimed to raise awareness and encourage engagement, while “Develop educational materials” represented 20.8% and focused on providing appealing resources. Partner engagement strategies followed, with “Maintain partner engagement” comprising 12.4% and “Distribute dissemination toolkit” 10.7%, both supporting ongoing collaboration and partner-led promotion. Fewer than 7% of strategies involved “Develop promotional messages,” “Build partnerships,” “Conduct partner educational meetings,” “Distribute educational materials,” and “Inform school leaders.”

“Promotion via mass media” was also the most intensive strategy, requiring 110 person-hours, followed by “Develop educational materials” with 48 person-hours. Although used less frequently, “Develop promotional messages” demanded 42 person-hours—comparable to “Develop educational materials”—while “Build partnerships” required 24 person-hours, more than “Distribute dissemination toolkit” at 12 person-hours. These variations in frequency and intensity reflected differences in the number of people involved and the time spent across activities, as detailed in Supplementary File 2. Simpler activities, such as emailing partner organizations to distribute the toolkit, typically involved one team member and took around 0.25 h. In contrast, more complex activities, such as developing social-media content for Instagram, Facebook, and TikTok, required up to six team members and could take up to 5 h to complete.

### Temporality: dissemination strategies type, frequency, and intensity over time

**Figure 2.**
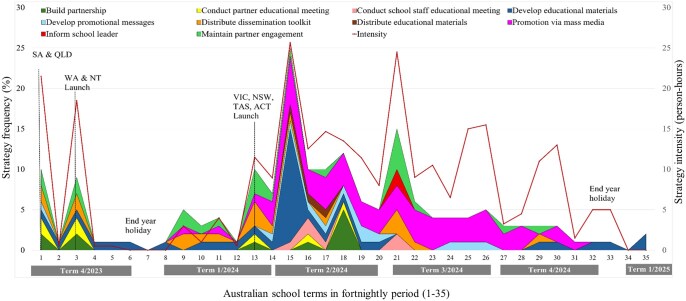
Temporality**—**type, frequency, and intensity of dissemination strategies used over time. NSW, New South Wales; VIC, Victoria; QLD, Queensland; WA, Western Australia; SA, South Australia; TAS, Tasmania; ACT, Australian Capital Territory; NT, Northern Territory.

In terms of temporality, Figure 2 shows that the overall strategy frequency and intensity fluctuated over time. There were several spikes occurring around state launches and at the beginning of school terms, with the highest peak observed at the start of Term 2 in 2024, right after the final launch in Victoria and other states and territories. Strategy type changed over time, with the *TransformUs* team predominantly employing strategies related to engaging partner organizations in dissemination during the launch period. These included “Conduct partner educational meeting” during launches and presentations to partners, “Maintain partner engagement” through follow-up meetings postlaunch, and “Distribute dissemination toolkit” during partner meetings. However, after the final launches covering Victoria, the Australian Capital Territory, New South Wales, and Tasmania, a broader range of strategies targeting school staff emerged. These included “Promotion via mass media,” such as social media posts and editorials in teacher magazines; “Conduct teacher educational meeting,” including online teacher professional development webinars; and “Distribute educational materials.” There was a notable increase in message and material development postlaunch, particularly in educational materials.

### Dissemination pathways

**Figure 3.**
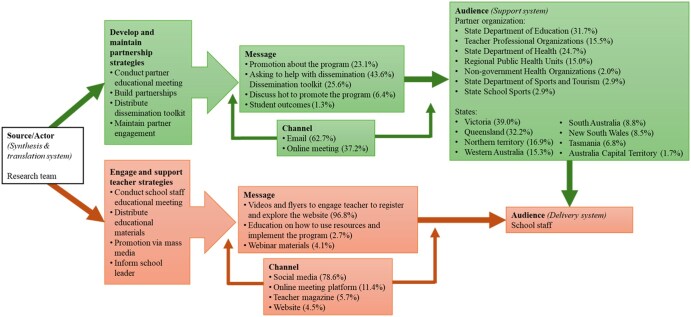
Overview of dissemination pathways of *TransformUs Secondary* initiative from *TransformUs Team* to partner organization and school staff: adapted from Brownson *et al.*’s Dissemination Model [6]. Percentages are based on the frequency of messages/channels used and target audiences approached for each dissemination strategies. The orange color represents direct dissemination (54.3%), while the green color represents indirect dissemination (45.7%). This model integrates the Interactive Systems Framework [23] to define dissemination roles. The *TransformUs* research team serves as the Synthesis and Translation System, developing and packaging initiative materials. Partner organizations represent the Support System, promoting the initiative and providing training. School staff and school leaders act as the Delivery System, implementing the initiative in school settings.

Figure 3 shows that the *TransformUs* team, as the source within the Synthesis and Translation System, used both direct and indirect pathways to reach school staff and school leaders in the delivery system, as well as partner organizations in the support system. Among the 10 identified strategies, two were classified as development strategies used in preparation before reaching the target audiences and were therefore excluded from the audience pathway illustration. The remaining eight were engagement strategies involving message/material distribution and efforts to enhance audience awareness and engagement. These eight strategies were grouped into two overarching categories. Four strategies aimed to engage partner organizations within the support system: “Conduct partner educational meeting,” “Build partnership,” “Distribute dissemination toolkit,” and “Maintain partner engagement.” The remaining four targeted school staff within the delivery system: “Promotion via mass media,” “Conduct teacher educational meeting,” “Distribute educational materials,” and “Inform school leader.” Most strategies, accounting for 54.3% of the total, followed a direct pathway. Among these direct strategies, the most common involved targeting school staff through social and mass media, which represented 78.6% of direct pathways and aimed to promote registration and website use.

During the dissemination period, all Australian states and territories were targeted, with the largest proportions of activities occurring in Victoria at 39.0%, Queensland at 32.2%, and South Australia at 28.8%. Indirect dissemination reached school staff via 16 partner organizations, primarily within the education sector, including departments of education across states, which accounted for 31.7%, and professional teaching associations (e.g. subject principal association, physical education teacher association) at 15.5%. The health sector comprised departments of health across states at 24.7%, regional public health units at 15.0%, and nongovernment health organizations at 2.0%. The sports and recreation sector included state departments of sport and recreation and state school sports, each contributing 2.9%. Partners were engaged mainly through email, which represented 62.7% of communication, and online meetings, which accounted for 37.2%, to request dissemination support.

## Discussion

This study described the national dissemination of the *TransformUs Secondary* initiative over a 16-month period, including the classification of strategies by type, frequency, intensity, temporality, and pathway. Using the SISTER taxonomy [13] and Leeman *et al.*’s framework [11], we identified 10 discrete strategies and two overarching strategy types targeting multiple audiences across system levels. The variety of strategy types reflects those found across studies, which typically include knowledge synthesis (developing messages and materials), communication (distributing messages and materials), interaction, and persuasion (educating and engaging the audience) [13]. Dissemination strategies were operated across multiple system levels and engaged both partner organizations and schools. Dissemination strategies increased in variation and intensity following the final state/territory launches.

Across the 16-month dissemination period, the most used strategy was to raise school staff awareness and foster their engagement, such as promotion via social and mass media and educational material development and distribution. This reflects the critical role school staff play in adopting school-based programs and aligns with evidence that staff engagement is a key facilitator of program uptake [24]. Dissemination through trusted channels (e.g. teacher social media, teacher magazine, and education conferences) while promoting program benefit and curriculum alignment is essential to motivate program adoption [25]. Educational material development may further support dissemination, with improvements to website-based materials (e.g. active break resources) enhancing usability [26, 27]. Website-based dissemination has been highlighted as valuable for increasing reach, fostering connection, and supporting enjoyable activity experiences, while simultaneously providing scalable infrastructure for sustained access [28]. However, evidence increasingly suggests that passive dissemination, such as simply providing materials on a website, is often insufficient to promote meaningful uptake [6]. Instead, strategies that actively engage implementers and build their capacity, such as interactive workshops, are more likely to positively influence program adoption [29, 30]. It underscores the importance of actively engaging a range of implementers as successful factors for scale-up [31], with teacher-focused dissemination strategies forming a critical entry point. Future studies should focus on dissemination strategies that not only provide access to materials but also actively engage and build teachers’ skills to promote meaningful program adoption.

The other commonly used strategies focused on actively engaging the 16 state partner organizations across the education, health, and sports sectors. This is consistent with evidence that one-third of partners in health promotion programs come from outside the health sector [12], highlighting the value of cross-sector engagement when navigating interconnected policies and systems [32]. These partners, which included policymakers and practitioners, were key dissemination audiences due to their system-level influence, positioning them as intermediaries [6]. This strategy reflects a common approach in Australia, where school-based programs such as *PA4E1* [33] and *RT for Teens* (Resistance Training for Teens) [33] partnered with the state Department of Education to facilitate dissemination. Aligning the initiative with partners’ priorities, providing dissemination toolkits, and demonstrating support for school staff implementation are critical, as partners are more influenced by policy alignment, feasibility [34], and strategies that enhance implementer capacity [29]. In *TransformUs Secondary*, partner engagement was maintained beyond launch periods, which might be important for sustaining visibility and reinforcing trust. Previous research shows that sustained partner engagement is vital during dissemination and scale up for maintaining trust and commitment [34, 35]. Future studies should explore the most effective strategies to engage partner organizations in dissemination and examine how their efforts influence reaching the schools and teachers.

Dissemination strategies varied in type, frequency, and intensity over the 16-month scale-up, with spikes around launch periods and school term starts. Launch spikes focused on strategies related to partner engagement, while postlaunch spikes targeted school staff engagement and educational material development. It suggests a strategic shift from building system-level support to fostering material provision and school level engagement. Over time, the temporality of strategies aligned with school staff’s planning cycles (start of school terms) to enhance responsiveness. This pattern reflects the nonlinear, phased nature of scale-up, influenced by milestones, readiness, and context [36]. Furthermore, strategies differed in intensity, shown by variations in time and actors involved, consistent with Powell *et al.* [37]. For example, message and material development strategies for school staff were resource-intensive, compared to simpler strategies like distributing a dissemination toolkit to partner organizations via email. It is important to note that the intensity measures reported here may not fully capture the total time required by the team to complete each activity. For example, sending an email was recorded as 0.25 h, but this does not account for additional tasks such as preparing the message, compiling attachments, or generating contact lists.

Furthermore, some activities may have required more time or personnel due to unfamiliarity with tasks, coordination challenges, or learning processes, particularly early in the program. Consequently, the recorded person-hours may over- or underestimate the actual effort associated with each dissemination strategy. While we did not evaluate the effectiveness of different intensities, this documentation provides a starting point for future studies to consider resource allocation and cost-effectiveness when designing dissemination strategies [38]. For example, our experience indicates that a minimum team of ∼7 members, with expertise in web management, resource development, media and technical support, and partner engagement, was required to effectively deliver and monitor dissemination at scale. In terms of strategy use overtime, findings from previous research show that during scale-up, dissemination channels become more diverse and intense, with direct outreach to delivery settings increasing by 16% [12]. As programs move from initial rollout to wider adoption, strategies typically shift from broad to more targeted efforts, decreasing in frequency but increasing in intensity [35]. This highlights how scale-up demands more complex and resource-heavy dissemination as it progresses. Future studies that examine the effectiveness of dissemination strategies should consider timing according to the school context (e.g. school calendar), explore how patterns vary over time, and measure the intensity of strategy used.

Consistent with Leeman *et al.*’s definition of dissemination strategies as deliberate actions to promote awareness and uptake across audiences [11], this study identified both direct and indirect pathways used to reach different target audiences. Identifying target audiences was essential to ensure that dissemination channels—such as emails, newsletters, and social media posts for school staff, and presentations or partner-led toolkits and meetings for partner organizations—were relevant and effectively delivered [6, 30]. It underscores the importance of disseminating through multiple, audience-preferred channels [39], while tailoring messages (e.g. using narratives, data, or visualizations) to audiences’ knowledge, values, and goals [40].

Evidence from a systematic review suggests that tailored and targeted channels and messages can influence not only knowledge but also decision-making practices among the target audience [29]. These findings highlight that dissemination pathway is rarely a straightforward process flowing directly from source to channel to audience as depicted in dissemination model [6]. In practice, dissemination requires simultaneous engagement with both the target audience (school staff) and the broader network of stakeholders involved in scale-up. It aligns with dissemination frameworks such as the Health Promotion Research Center dissemination framework [41] and models used in school mental health initiatives [30], in which system-level actors (e.g. policy makers and partner organizations) act as intermediaries to reach schools. Future research should empirically test the effectiveness of these dissemination pathways and examine how intermediary organizations influence uptake in schools, particularly given that stakeholders from different sectors, types, and structures may vary in their capacity to support dissemination efforts.

This study is the first to systematically document national dissemination strategies type, frequency, intensity, and pathways for a national scale-up, whole-of-school physical activity and sedentary behavior intervention. Previous school-based physical activity programs like *The Sports, Play, and Active Recreation for Kids Physical Education* (*SPARK PE)*, *The National Football League* (*NFL Play 60)*, and *RT for Teens*, briefly described dissemination activities such as conferences, social media, or school staff training [33, 42, 43], limiting understanding of how dissemination unfolded in practice and how different strategies contributed to varying outcomes. Thus, more scaled interventions that explain the dissemination rationale and structure are needed for improved interpretability, replicability and determination of effectiveness. This study also described the dissemination pathway, highlighting the need to tailor messages and channels to engage multilevel audiences. The *TransformUs* team itself acted as a dissemination vehicle, suggesting that the research team’s role could also be conceptualized as a strategy. Importantly, this study tracked frequency and intensity of strategies overtime, offering insights into the strategic sequencing and adaptations across the scale-up lifecycle. A limitation, however, is that strategies often overlapped and were difficult to categorize distinctly. This also meant that aligning dissemination strategies with adoption and implementation outcomes was not possible. Other limitations included reliance on recall that may miss informal dissemination, and dissemination and estimated person-hours may not accurately reflect actual effort. Additionally, partner-led indirect dissemination to schools was not systematically captured.

## Conclusion

Mapping and specifying dissemination strategies, along with mapping dissemination pathways offers valuable understanding of the complex, nonlinear nature of scaling up initiatives in real-world contexts. This approach enhances clarity and replicability which are essential to inform future dissemination strategies and scale-up.

## Supplementary Material

ibaf089_Supplementary_Data

## Data Availability

The data that support the findings of this study are available from the corresponding author, A.F., upon reasonable request.

## Welfare of Animals

This article does not contain any studies with animals performed by any of the authors.

## Transparency Statement

1. Study Registration: This study was prospectively registered with the Australian New Zealand Clinical Trials Registry (ANZCTR), registration number ACTRN12622000600741 (https://www.anzctr.org.au/Trial/Registration/TrialReview.aspx?id=382976)
2. Analytic Plan Pre-Registration: The analysis plan was pre-registered as part of the trial documentation on the ANZCTR under the ID ACTRN12622000600741
3. Analytic Code Availability: Analytic code used to conduct the analyses presented in this study are not available in a public archive. They may be available by emailing the corresponding author, AF.
4. Materials Availability: The activity log used to document dissemination activities is included as a supplementary file.
