# Supplementary material for: Mapping the type, frequency, intensity, temporality, and pathways of dissemination strategies during the national scale-up of TransformUs Secondary
Source: Transl Behav Med. 2026 Jan 13;16(1):ibaf089. doi: 10.1093/tbm/ibaf089 (PMC12803782; doi:10.1093/tbm/ibaf089)
Supplement: ibaf089_Supplementary_Data [file ibaf089_supplementary_data.zip › Supplementary File 1.docx]

Supplementary File 1. Dissemination Activity Log

| Dissemination Activity Log (with *examples*) | | | | | | | | | |
| --- | --- | --- | --- | --- | --- | --- | --- | --- | --- |
| Date | Activity name | Purpose | Duration | *TransformUs* Team involved | Target audiences/ attendees | Details of activity | Media/channel used | Message delivered | Output (if any) |
| *03/10/2023* | *Website* | *Inform* | *1 hours* | *4 persons* | *South* | *An online* | *Online* | *Promotion* | *Dissemination* |
|  | *launch* | *program* |  |  | *Australia* | *webinar to* | *meeting* | *about the* | *toolkit sent* |
|  | *for South* | *availability* |  |  | *key* | *inform* |  | *program,* |  |
|  | *Australia* | *and its* |  |  | *partners* | *partners* |  | *asking to* |  |
|  |  | *benefits,* |  |  |  | *about the* |  | *help with* |  |
|  |  | *build* |  |  |  | *launched* |  | *dissemination* |  |
|  |  | *partnerships* |  |  |  | *TransformUs* |  |  |  |
|  |  | *for* |  |  |  | *Secondary* |  |  |  |
|  |  | *dissemination* |  |  |  | *dashboard,* |  |  |  |
|  |  | *support.* |  |  |  | *presented by* |  |  |  |
|  |  |  |  |  |  | *the team.* |  |  |  |
| *27/03/2024* | *Social* | *Promote the* | *0.5 hours* | *3 persons* | *School* | *Post* | *Social media* | *Introducing* | *Social media* |
|  | *media* | *program to* |  |  | *staff* | *engaging* |  | *new website* | *followers and* |
|  | *post* | *encourage* |  |  |  | *content* |  | *content,* | *likes* |
|  |  | *school staff* |  |  |  | *(videos,* |  | *explaining* |  |
|  |  | *registration* |  |  |  | *flyers) on* |  | *website* |  |
|  |  | *and resource* |  |  |  | *Instagram,* |  | *features* |  |
|  |  | *use.* |  |  |  | *Facebook, X,* |  |  |  |
|  |  |  |  |  |  | *TikTok,* |  |  |  |
|  |  |  |  |  |  | *LinkedIn* |  |  |  |
