# Supplementary material for: Mapping the type, frequency, intensity, temporality, and pathways of dissemination strategies during the national scale-up of TransformUs Secondary
Source: Transl Behav Med. 2026 Jan 13;16(1):ibaf089. doi: 10.1093/tbm/ibaf089 (PMC12803782; doi:10.1093/tbm/ibaf089)
Supplement: ibaf089_Supplementary_Data [file ibaf089_supplementary_data.zip › Supplementary File 2.docx]

Supplementary File 2. Categorization of Dissemination Strategies

| No. | Strategy name | Definition for  *TransformUs Secondary* | Unique activity | Persons (median,  min-max) | Hours (median,  min-max) | Channel | Message | Target |
| --- | --- | --- | --- | --- | --- | --- | --- | --- |
| 1 | Develop promotional messages^a^ | Coordinated efforts to frame and craft messages for promoting the  program | Social media messaging, video production, social media planning  meetings | 6  (1-6) | 0.8  (0.5-3) | N/A | N/A | N/A |
| 2 | Develop educational materials^c^ | Improve learning resources and website content to make them more  engaging | Website development and updates, Active Breaks update | 1  (1-5) | 0.5  (0.5-5) | N/A | N/A | N/A |
| 3 | Conduct partner educational meeting^b^ | Conducting formal meetings to educate partners about  the program | Presentations to partners, website state launch | 4  (2-4) | 1  (0.5-1) | Online meeting | Promotion about the program and asking to help with  dissemination | Partner organizations  Partner organizations |
| 4 | Distribute dissemination toolkit^d^ | Distributing dissemination tools to  partners to | Dissemination toolkit distribution | 1  (1-2) | 0.3  (0.3-2) | Email | Dissemination toolkit |  |

| No. | Strategy name | Definition for  *TransformUs Secondary* | Unique activity | Persons (median,  min-max) | Hours (median,  min-max) | Channel | Message | Target |
| --- | --- | --- | --- | --- | --- | --- | --- | --- |
|  |  | help them |  |  |  |  |  | Partner organizations |
|  |  | disseminate |  |  |  |  |  |  |
|  |  | the program |  |  |  |  |  |  |
| 5 | Build | Develop | Partner | 2 | 0.7 | Online | Promotion |  |
|  | partnerships^b^ | collaborations | engagement and | (1-4) | (0.5-1) | meeting, | about the |  |
|  |  | with partner | alignment |  |  | email | program and |  |
|  |  | organizations | through |  |  |  | asking to help |  |
|  |  | to support | launches, partner |  |  |  | with |  |
|  |  | dissemination | presentations, |  |  |  | dissemination |  |
|  |  |  | and outreach to |  |  |  |  |  |
|  |  |  | potential new |  |  |  |  |  |
|  |  |  | partners |  |  |  |  |  |
| 6 | Maintain partner | Sustain | Post-launch | 1 | 0.3 | Email, | Asking to | Partner organizations |
|  | engagement ^d^ | partner | partner meeting, | (1-2) | (0.3-4) | online | help with |  |
|  |  | engagement | identification of |  |  | meeting | dissemination, |  |
|  |  | to support | partners’ |  |  |  | discuss how |  |
|  |  | dissemination | promotion |  |  |  | to promote |  |
|  |  |  | opportunity |  |  |  | the program, |  |
|  |  |  | post-launch |  |  |  | students’ |  |
|  |  |  |  |  |  |  | outcome |  |
| 7 | Conduct school | Conducting | Online | 1 | 0.7 | Online | Promotion | School staff |
|  | staff | formal | professional | (1-2) | (0.5-0.7) | meeting | about the |  |
|  | educational | meetings to | learning, |  |  |  | program, |  |
|  | meeting^b^ | educate | presentations to |  |  |  | engage school |  |
|  |  | school staff | school staff |  |  |  | staff to |  |
|  |  | about the |  |  |  |  | explore and |  |
|  |  | program |  |  |  |  | use resources |  |

| No. | Strategy name | Definition for  *TransformUs Secondary* | Unique activity | Persons (median,  min-max) | Hours (median,  min-max) | Channel | Message | Target |
| --- | --- | --- | --- | --- | --- | --- | --- | --- |
|  |  |  |  |  |  |  | from the  website |  |
| 8 | Distribute educational materials^c^ | Distributing resources post- educational  meeting | Webinar material upload | 1  (1-1) | 0.5  (0.5-0.5) | Website | Webinar materials | School staff |
| 9 | Promotion via mass media^c^ | Utilizing widespread platforms (e.g., social media, teacher magazine) for  promotion | Social media posting, social media group engagement, school staff magazine editorial | 3  (1-3) | 0.5  (0.5-4) | Social media, teacher magazine | Engage school staff to register and explore the website | School staff |
| 10 | Inform school leaders^b^ | Informing school leaders who can help advocate for and promote the program | Presentations to school leaders | 2  (2-2) | 0.5  (0.5-0.5) | Online meeting | Promotion about the program and engage school staff to register and explore the  website | School staff |

^a^ Strategies categorized according to Leeman et al.'s framework

^b^ Strategies classified under Cook et al.'s SISTER taxonomy

^c^ Strategies that overlap between both frameworks

^d^ Strategies not indexed in either framework
